# ANGPTL4 silencing via antisense oligonucleotides reduces plasma triglycerides and glucose in mice without causing lymphadenopathy

**DOI:** 10.1016/j.jlr.2022.100237

**Published:** 2022-06-03

**Authors:** Mingjuan Deng, Elda Kutrolli, Anne Sadewasser, Sven Michel, Masoumeh Motamedi Joibari, Frank Jaschinski, Gunilla Olivecrona, Stefan K. Nilsson, Sander Kersten

**Affiliations:** 1 Nutrition, Metabolism and Genomics Group, Division of Human Nutrition and Health, Wageningen University, Wageningen, the Netherlands; 2 Lipigon Pharmaceuticals AB, Umeå, Sweden; 3 Secarna Pharmaceuticals GmbH & Co. KG, Planegg, Germany; 4 Department of Medical Biosciences, Umeå University, Umeå, Sweden

**Keywords:** adipose tissue, liver, lipase/lipoprotein, lipoproteins/metabolism, triglycerides, angiopoietin-like 4, antisense oligonucleotides, high-fat diet, fasting, ANGPTL4, angiopoietin-like 4, ASCVD, atherosclerotic cardiovascular disease, ASO, antisense oligonucleotide, HFD, high-fat diet, LNA, locked nucleic acid, TG, triglyceride, TLR9, Toll-like receptor 9

## Abstract

Angiopoietin-like 4 (ANGPTL4) is an important regulator of plasma triglyceride (TG) levels and an attractive pharmacological target for lowering plasma lipids and reducing cardiovascular risk. Here, we aimed to study the efficacy and safety of silencing ANGPTL4 in the livers of mice using hepatocyte-targeting GalNAc-conjugated antisense oligonucleotides (ASOs). Compared with injections with negative control ASO, four injections of two different doses of ANGPTL4 ASO over 2 weeks markedly downregulated ANGPTL4 levels in liver and adipose tissue, which was associated with significantly higher adipose LPL activity and lower plasma TGs in fed and fasted mice, as well as lower plasma glucose levels in fed mice. In separate experiments, injection of two different doses of ANGPTL4 ASO over 20 weeks of high-fat feeding reduced hepatic and adipose ANGPTL4 levels but did not trigger mesenteric lymphadenopathy, an acute phase response, chylous ascites, or any other pathological phenotypes. Compared with mice injected with negative control ASO, mice injected with ANGPTL4 ASO showed reduced food intake, reduced weight gain, and improved glucose tolerance. In addition, they exhibited lower plasma TGs, total cholesterol, LDL-C, glucose, serum amyloid A, and liver TG levels. By contrast, no significant difference in plasma alanine aminotransferase activity was observed. Overall, these data suggest that ASOs targeting ANGPTL4 effectively reduce plasma TG levels in mice without raising major safety concerns.

Mounting evidence from human genetic studies suggests that elevated plasma triglyceride (TG) levels are a causal and independent risk factor for atherosclerotic cardiovascular disease (ASCVD) (1). How plasma TG can be targeted to lower the risk of ASCVD is the subject of intense investigations. TGs are present in blood plasma as part of TG-rich lipoproteins, consisting of chylomicrons and VLDLs. The hydrolysis of plasma TG is catalyzed by LPL, an enzyme secreted by adipocytes, (cardio)myocytes, and macrophages (2). By mediating intravascular lipolysis, LPL plays a vital role in regulating plasma TG levels and in ensuring that tissues can acquire plasma lipids for storage or use as fuel (2, 3). The activity of LPL is regulated post-translationally by several proteins including the apolipoproteins C1, C2, C3, A5, and E. In addition, LPL activity is governed by three members of the angiopoietin-like protein family (ANGPTL): ANGPTL3, ANGPTL4, and ANGPTL8 (4).

ANGPTL3, ANGPTL4, and ANGPTL8 are expressed in a tissue-specific manner and are highly sensitive to nutritional cues (5, 6, 7, 8, 9). ANGPTL3 is exclusively secreted by the liver and cooperates with ANGPTL8 in the fed state to inhibit LPL activity in oxidative tissues such as the heart and brown fat (5, 10). Human genetic studies have shown that loss-of-function variants in ANGPTL3 are associated with reduced plasma TG and LDL-C levels and a reduced risk of ASCVD (11, 12, 13). These findings have spurred the development of novel therapeutics that inactivate ANGPTL3, which include the recently approved evinacumab for the treatment of homozygous familial hypercholesterolemia (11, 14). In contrast to ANGPTL3, ANGPTL4 is produced by multiple cells and tissues, including the liver, adipose tissue, heart, and macrophages, and mainly plays a role in LPL regulation in the fasted state (15, 16). Mechanistic studies have shown that ANGPTL4 promotes the unfolding of LPL, which triggers the cleavage of LPL by proprotein convertase subtilisin/kexin type 3 and its subsequent intracellular degradation, thereby reducing the amount of LPL present on the capillary endothelium (17, 18, 19, 20, 21). The importance of ANGPTL4 in the regulation of plasma TG in humans is supported by human genetics indicating that loss-of-function variants in ANGPTL4 are associated with decreased plasma TG levels and a reduced risk of ASCVD (22, 23, 24, 25, 26).

The preponderance of genetic evidence coupled with a detailed understanding of its mechanism of action makes ANGPTL4 a highly attractive pharmacological target for plasma TG lowering. However, the development of anti-ANGPTL4 strategies has been hampered by the observation that whole-body inactivation of ANGPTL4 can lead to severe clinical problems in mice. Specifically, mice deficient in ANGPTL4 develop mesenteric lymphadenopathy, a massive acute phase response, intestinal fibrosis, chylous ascites, fibrinopurulent peritonitis, weight loss, and ultimately die when fed a diet high in saturated fatty acids (27, 28). Similar pathological changes were observed in mice injected with anti-ANGPTL4 antibodies (29). Furthermore, several female monkeys treated with ANGPTL4-inactivating antibodies showed lymphadenopathy characterized by lipid accumulation in the mesenteric lymph nodes (22). Whether whole-body inactivation of ANGPTL4 might trigger similar pathological features in humans is unclear. So far, there has not been any indication that homozygous ANGPTL4 loss-of-function carriers exhibit any of the above features or have a higher chance of abdominal lymphatic disorders (22). Nevertheless, because of lingering safety concerns associated with whole-body ANGPTL4 inactivation, the tissue-specific inactivation of ANGPTL4 merits further investigation.

Two tissues characterized by a high level of ANGPTL4 expression are the adipose tissue and liver. Previous work in mice has demonstrated that depletion of ANGPTL4 in adipocytes significantly reduces plasma TG levels (30, 31). Unfortunately, in humans, it is currently not possible to inactivate genes specifically in adipocytes. Concerning the liver, an early study found that liver-specific overexpression of ANGPTL4 elevates plasma TG levels (32). More recently, it was shown that liver-specific deficiency of ANGPTL4 did not influence plasma TG levels after a 6-h fast but significantly reduced plasma TG levels in the prolonged fasted state (33, 34). In addition, silencing of ANGPTL4 using hepatocyte-targeting GalNAc-conjugated antisense oligonucleotides (ASOs) significantly decreased plasma TG levels in overnight fasted mice (33). Collectively, these data show that hepatic ANGPTL4 raises plasma TG in the (prolonged) fasted state.

In the present study, we used ASOs to silence hepatic ANGPTL4. ASOs consist of modified DNA complementary to the target RNA. In the nucleus, they bind to the target mRNA or pre-mRNA and recruit the nuclear enzyme RNAseH, which cleaves the target RNA, leading to reduced gene expression. To protect the ASO from nuclease-mediated degradation, ASOs have a phosphorothioate instead of a phosphodiester backbone. To increase affinity to the target, the flanks contain locked nucleic acid (LNA)-modified nucleotides (35, 36). In vitro, LNA-ASOs achieve specific target knockdown in many different cell types without special delivery reagents. In vivo, unformulated LNA-ASOs induce knockdown of gene expression in different organs and tissues after systemic delivery. Conjugation of triantennary *N*-acetyl-galactosamine (GalNAc_3_) to ASOs has been shown to increase the productive delivery of ASOs to the liver by binding to the asialoglycoprotein receptor, which is almost exclusively expressed on hepatocytes (37, 38, 39). Accordingly, GalNAc_3_ conjugation to ASOs is expected to result in a substantial increase in potency for hepatocyte-produced target RNAs.

Here, we aimed to study the efficacy and safety of silencing ANGPTL4 in livers of mice using hepatocyte-targeting GalNAc-conjugated ASOs.

## Materials and methods

### Selection of mouse Angptl4-specific ASO

Mouse *Angptl4*-specific ASOs used for screening were synthesized by Eurogentec (Cologne, Germany). After identification of the most suitable candidate, ANGPTL4 ASO (+C∗+T∗+C∗A∗T∗G∗T∗T∗A∗G∗G∗T∗A∗G∗+G∗+T∗+T) and negative control oligonucleotide negative control ASO (Neg-Ctrl ASO) (+C∗+G∗+T∗T∗T∗A∗G∗G∗C∗T∗A∗T∗G∗T∗A∗+C∗+T∗+T, sequence derived from the literature (40) (+ indicating a LNA modification and ∗ a phosphorothioate modification) were purchased from Axolabs (Kulmbach, Germany) for subsequent in vivo studies. ASOs were purified by reverse-phase HPLC and subsequently lyophilized. For the in vitro studies, the ASOs were dissolved in diethyl-pyrocarbonate-treated H_2_O to a concentration of 1 mM.

For the in vivo studies, *N*-acetylgalactosamine (GalNac) was conjugated to the ANGPTL4 and Neg-Ctrl ASOs via aminohexyl linker. The ASOs were diluted in saline to a concentration of 0.5 mg/ml and kept at −20°C before performing subcutaneous injections.

### IC_50_ determination

About 15,000 primary mouse hepatocytes (Lonza) per well were seeded in BioCoat Collagen I 96-well flat-bottom plates (catalog no.: A11428-03; Thermo Fisher Scientific) in 100 μl Hepatocyte Plating Medium (catalog no.: MP250; Lonza). Supernatant was removed 4–6 h after seeding, and ASOs were added at indicated concentrations (5,000, 1,000, 200, 40, 8, and 1.6 nM) diluted in 100 μl Maintenance Medium (catalog no.: MM250; Lonza). Cells were cultured for 3 days at 37°C. Afterward, the cells were lysed, and mRNA levels were measured according to the manufacturer's instructions, using the QuantiGene Singleplex Gene Expression Assay (catalog no.: QS0011; Thermo Fisher Scientific) and the following probesets: murine ANGPTL4 (catalog no.: SB-16744-02; Thermo Fisher Scientific) and HPRT1 (catalog no.: SB-15463; Thermo Fisher Scientific). Values were normalized to the housekeeping gene HPRT1. IC_50_ values were calculated using Prism 6 (GraphPad Software, Inc). Data are represented as the mean of triplicate wells ± SD relative to untreated cells (set as 1).

### Toll-like receptor 9 reporter assay

The Toll-like receptor 9 (TLR9) reporter gene assay was performed as previously described (41). Briefly, stably transfected human embryonic kidney cells expressing a mouse TLR9 NF-κB luciferase reporter plasmid were treated with ODN1668 (catalog no.: tlrl-1668; InvivoGen) and ANGPTL4 ASO or Neg-Ctrl ASO at indicated concentrations (5,000, 1,000, 200, 40, 8, and 1.6 nM). Each condition was performed in triplicates. After 24 h, the cell supernatants were removed, and ONE-Glo EX reagent (50 μl, catalog no.: E8110; Promega) was added for cell lysis according to the manufacturer’s instructions. Luminescence was immediately measured at 560 nm. Data are represented as the mean of triplicate wells ± SD.

### Human liver and adipose tissue samples

Human liver and adipose tissue samples were acquired from the Mondial study and processed as previously described (42).

### Animal studies

Study 2 was carried out at the Umea Center for Comparative Biology and approved by the European Union (Directive 2010/63/EU) and the Swedish legislative authorities (Swedish Animal Protection Regulation [1988: 539]; Animal Protection Act [SFS 1988:534]; Swedish Agricultural Agency's regulations [L150]). Study 1 and 3 were carried out at the Centre for Small Animals, which is part of the Centralized Facilities for Animal Research at Wageningen University and Research (CARUS), and were approved by the Institutional Animal Care and Use Committee (AVD104002015236, 2016.W-0093.018, and 2016.W-0093.023).

#### Study 1

Male C57BL/6J mice were acquired from Jackson Laboratories (no. 000664) and further bred at the animal facility of Wageningen University for >10 generations. The mice were housed at two mice per cage at 21–22°C under specific pathogen-free conditions and according to a 6:00–18:00 day-night cycle. The experiment was run in two separate batches of 35 mice spaced 5 weeks apart. In the first batch, mice at 10–13 weeks of age were randomly assigned by cage to Neg-Ctrl ASO (1.25 mg/kg, *n* = 12), low-dose ANGPTL4 ASO (0.625 mg/kg, *n* = 12), or high-dose ANGPTL4 ASO (1.25 mg/kg, *n* = 11). The ASOs were injected subcutaneously twice a week at the indicated dose (dissolved in PBS, final volume of 100 μl) for a total of four injections. After 2 weeks, the mice were euthanized between 8.30 and 10.30 in the morning after a 24 h fast. In the second batch, mice at 10–14 weeks of age were randomly assigned by cage to Neg-Ctrl ASO (1.25 mg/kg, *n* = 12), low-dose ANGPTL4 ASO (0.625 mg/kg, *n* = 12), or high-dose ANGPTL4 ASO (1.25 mg/kg, *n* = 11). The ASOs were injected subcutaneously twice a week at the indicated dose (dissolved in PBS, final volume of 100 μl) for a total of four injections. After 2 weeks, the mice were euthanized between 8.30 and 10.30 in the morning in the ad libitum fed state. The mice were anesthetized with a mixture of isoflurane (1.5%), nitrous oxide (70%), and oxygen (30%), followed by the collection of blood by eye extraction into EDTA tubes. The mice were euthanized by cervical dislocation, after which tissues were excised and snap-frozen in liquid nitrogen.

One mouse in the 24 h-fasted high-dose ANGPTL4 ASO group and one mouse in the 24 h-fasted Neg-Ctrl ASO group were excluded from further analysis because of liver pathology (coarse liver).

#### Study 2

Male C57BL/6J mice were supplied by Charles River Laboratories, Inc ('s-Hertogenbosch, the Netherlands). All mice were housed in group cages under a 12 h light/12 h dark cycle. The mice were given ad libitum access to food and water. All mice received a high-fat diet (HFD; rodent diet with 60 kcal% fat; catalog no.: D12492; Research Diets, Inc, New Brunswick, NJ) for 3.5 months before the start of the study, and this diet was kept throughout the study.

The mice were randomly assigned to treatment with Neg-Ctrl ASO (1.25 mg/kg, *n* = 8) or ANGPTL4 ASO (1.25 mg/kg, *n* = 8). The mice received a subcutaneous injection (100 μl) of the assigned ASOs twice a week for a total of 40 injections. Bodyweight and food intake were monitored throughout the study. An intraperitoneal glucose tolerance test was carried out after 19 weeks. After 20 weeks, body composition was measured by echo MRI, and blood was collected through the tail vein after a 6-h fast. Immediately thereafter, the mice were euthanized by cervical dislocation, followed by harvesting of the organs. Tissues and organs were stored at −80°C for future analyses.

#### Study 3

Male C57BL/6J mice at 3–4 weeks of age were purchased from Envigo (Horst, the Netherlands). The mice were housed at two mice per cage at 21–22°C under specific pathogen-free conditions and according to a 6:00–18:00 day-night cycle. The mice were given ad libitum access to food and water. The mice were randomly assigned per cage to treatment with Neg-Ctrl ASO (1.25 mg/kg, *n* = 10), low-dose ANGPTL4 ASO (0.625 mg/kg, *n* = 10), or high-dose ANGPTL4 ASO (1.25 mg/kg, *n* = 10). At 11 weeks of age, the diet was changed from standard chow to a semipurified HFD (rodent diet with 45 kcal% fat; catalog no.: D12451i; Research Diets, Inc). From that moment, the mice received a subcutaneous injection (100 μl) of the assigned ASOs twice a week for a total of 40 injections. Bodyweight and food intake were monitored throughout the study. After 20 weeks, the mice were euthanized between 8.30 and 10.30 in the morning. The mice were anesthetized with a mixture of isoflurane (1.5%), nitrous oxide (70%), and oxygen (30%), followed by the collection of blood by eye extraction into EDTA tubes. The mice were euthanized by cervical dislocation, after which tissues were excised and snap-frozen in liquid nitrogen.

### Intraperitoneal glucose tolerance test

The mice fasted for 5 h after which blood was collected via tail bleeding for baseline blood glucose measurement. Next, the mice received an intraperitoneal injection of glucose at 2 g/kg lean body mass, followed by blood collection via tail bleeding at 5, 15, 30, 45, 60, and 120 min. Blood glucose was measured with a GLUCOFIX Tech glucometer and glucose sensor test strips (GLUCOFIX Tech, Menarini Diagnostics, Valkenswaard, the Netherlands).

### Fast protein liquid chromatography-based lipoprotein profiling

Size-exclusion chromatography was performed on a Superose 6 3.2/300 column (GE Healthcare Life Science) using 10 μl of plasma. The column was run in elution buffer A (150 mM NaCl, 10 mM Tris, 0.02% azide, and pH 7.4) at a rate of 4 ml/min. The TG concentration in the eluate was determined by glycerine phosphate oxidase peroxidase reagent.

### Quantification of plasma parameters

Blood samples were collected into EDTA-coated tubes and centrifuged at 4°C for 15 min at 2,655 *g*. Plasma was collected and stored at −80°C. The plasma concentration of various metabolites/enzymes was determined using specialized kits: TGs (LiquiColor; Human GmbH, Wiesbaden, Germany), cholesterol (DiaSys Diagnostics Systems GmbH, Holzheim, Germany), glucose (Diasys), nonesterified fatty acids (Instruchemie, Delfzijl, the Netherlands), and glycerol (Instruchemie), following the instructions of the manufacturer.

### Hematoxylin and eosin staining

Hematoxylin and eosin staining was performed on the mesenteric lymph nodes and livers of the mice. Lymph nodes and liver pieces were placed into plastic cassettes and immediately fixed in 4% paraformaldehyde. The tissues were processed and embedded into paraffin blocks. Thin sections of the blocks were made at 5 μm using a microtome and placed onto Superfrost glass slides followed by overnight incubation at 37°C. The tissues were stained in Mayer hematoxylin solution for 10 min and eosin for 10 s at room temperature with intermediate washings in ethanol. The tissues were allowed to dry at room temperature followed by imaging using a light microscope.

### Liver glycogen and TGs

For measurement of liver glycogen, liver pieces were dissolved in 10 volumes of 1 M NaOH and incubated at 55°C. After 1–2 h, an equal volume of 1 M HCl was added, followed by centrifugation for 5 min at 3,000 rpm. Subsequently, 5 μl of supernatant was added to 50 μl of amyloglucosidase (1000 U/ml in 0.2 M sodium acetate buffer; pH 4.8) and incubated for 2 h with shaking (700 rpm) at 42°C. After a short centrifugation, glucose was measured using a glucose assay (Sopachem, Ochten, the Netherlands).

Liver pieces of ∼50 mg were homogenized using a Tissue Lyser II (Qiagen, Hilden, Germany) to a 5% homogenate (m/v) in 10 mM Tris, 2 mM EDTA, 0.25 M sucrose, pH 7.5. The TG concentration was then quantified using TG LiquiColor Mono kit from HUMAN Diagnostics (Wiesbaden, Germany) according to the manufacturer’s instructions.

### RNA isolations and quantitative PCR

Mouse tissues were homogenized in TRIzol (Invitrogen) using the Qiagen TissueLyser II and stainless steel beads. Total RNA was isolated using the RNeasy Micro kit from Qiagen (Venlo, the Netherlands). Subsequently, 500 ng of RNA was used to synthesize complementary DNA using the iScript cDNA Synthesis kit (Bio-Rad Laboratories, Veenendaal, The Netherlands). mRNA levels of various genes were determined by reverse transcription-quantitative PCR using SensiMix (Bioline; GC Biotech, Alphen aan den Rijn, The Netherlands) on a CFX384 real-time PCR detection system (Bio-Rad Laboratories). The housekeeping gene *Cyclophilin* was used for normalization. Primers were synthesized by Eurogentec (Seraing, Belgium).

The following primers were used:

*Cyclophillin*, Forward: CAGACGCCACTGTCGCTTT, Reverse: TGTCTTTGGAACTTTGTCTGCAA;

*Angptl4*, Forward: CCCACGCACCTAGACAATGG, Reverse: TGGGAGTCAAGCCAATGAGC;

*Angptl8*, Forward: CTGACCCTGCTCTTTCACGG, Reverse: GCTCTGTCATAGAGGCCCAG;

*Angptl3*, Forward: CAGGACTTCAACGAAACATGGG, Reverse: GAGTATTCAACGTAGTGCTTGCT;

*Lpl*, Forward: GGGAGTTTGGCTCCAGAGTTT, Reverse: TGTGTCTTCAGGGGTCCTTAG;

*Lipc*, Forward: CCCTGGCATACCAGCACTAC, Reverse: CTCCGAGAAAACTTCGCAGATT;

*Saa2*, Forward: GCGAGCCTACACTGACATGA, Reverse: TTTTCTCAGCAGCCCAGACT;

*Cd68*, Forward: CCAATTCAGGGTGGAAGAAA, Reverse: CTCGGGCTCTGATGTAGGTC;

*Ccl2*, Forward: CCCAATGAGTAGGCTGGAGA, Reverse: TCTGGACCCATTCCTTCTTG;

*Adgre1*, Forward: CTTTGGCTATGGGCTTCCAGTC, Reverse: GCAAGGAGGACAGAGTTTATCGTG;

*Lcn2*, Forward: TGGAAGAACCAAGGAGCTGT, Reverse: GGTGGGGACAGAGAAGATGA;

*Cxcl2*, Forward: CCAACCACCAGGCTACAGG, Reverse: GCGTCACACTCAAGCTCTG.

### Protein isolations and Western blot

Liver and adipose samples were lysed in RIPA lysis and extraction buffer (25 mM Tris-HCl [pH 7.6], 150 mM NaCl, 1% NP-40, 1% sodium deoxycholate, and 0.1% SDS; Thermo Fisher Scientific) with protease and phosphatase inhibitors (Roche). Following homogenization, lysates were placed on ice for 30 min and centrifuged two or three times at 13,000 *g* for 10 min at 4°C to remove fat and/or cell debris. Protein concentration was determined using a Pierce BCA kit, and equal amounts of protein were diluted with 4× Laemmli sample buffer (Bio-Rad). Protein lysates (10–30 μg of protein per lane) were loaded on an 8–16% gradient Criterion gel (Bio-Rad) and separated by SDS gel electrophoresis. Proteins were transferred to a polyvinylidene difluoride membrane using a Transblot Turbo System (Bio-Rad). Membranes were probed with a goat anti-mouse LPL antibody (43), a rabbit anti-mouse heat shock protein 90 antibody (catalog no.: 4874S; Cell Signaling), a rat anti-mouse ANGPTL4 antibody (Kairos 142-2; Adipogen), a rabbit anti-mouse ANGPTL4 antibody (catalog no.: 742; home-made) (44), a rabbit anti-human ANGPTL4 antibody (catalog no.: 1187; home-made), and a mouse anti-human LPL antibody 88b8 (45) at 1:5,000 (LPL), 1:2,000 (heat shock protein 90), or 1:1,000–2,000 (ANGPTL4) dilutions. Secondary antibodies were rabbit anti-mouse (1:5,000 dilution; catalog no.: G21040; Thermo Fisher Scientific), rabbit anti-goat (1:5,000 dilution; catalog no.: AP106P; Sigma-Aldrich), and goat anti-rabbit (1:5,000 dilution; catalog no.: AP187P; Sigma-Aldrich). Blocking and incubations with primary and secondary antibodies were done in Tris-buffered saline, pH 7.5, with 0.1% Tween-20 and 5% (w/v) skimmed milk. In between, membranes were washed in Tris-buffered saline with Tween-20. Primary antibodies were applied overnight at 4°C, and secondary antibody was applied for 1 h at room temperature. Blots were visualized using the ChemiDoc MP system (Bio-Rad) and Clarity ECL substrate (Bio-Rad).

### LPL activity assay

LPL activity was measured as previously described (31, 46). Frozen tissue samples were minced using surgical scissors and resuspended in LPL assay buffer (25 mM NH_4_Cl, 5 mM EDTA, 0.01% SDS, 45 U/ml heparin, 0.05% Zwittergent® 3–14 Detergent [catalog no.: 693017; Sigma-Aldrich]) containing protease inhibitor (Roche). The tissue lysate was then vortexed and incubated on ice for 30 min with intermittent disruption with surgical scissors. The lysate was then centrifuged at 15,000 *g* and 4°C for 15 min. Protein concentrations were equalized by using a Pierce BCA kit (Thermo Fisher Scientific) before assay activity. Supernatants were combined with the assay buffer (0.6 M NaCl, 80 mM Tris-HCl, pH 8, 6% fatty-acid free BSA, and 1% of the EnzChek lipase fluorescent substrate [catalog no.: E33955; Thermo Fisher Scientific]) in 96-well black clear-bottom plates. Fluorescence was measured from technical duplicates of each lysate (40 min, 37°C) on a Spectra Max i2 plate reader (Molecular Devices). Relative lipase activity was determined by calculating the linear slope of the curve and subtraction of background (assay buffer only) slope readings.

### Statistics

Statistical analysis was carried out in GraphPad using Student’s *t*-test, one-way ANOVA followed by Tukey’s multiple comparisons test, or two-way ANOVA followed by Tukey’s multiple comparisons test. *P* < 0.05 was considered statistically significant.

## Results

### High levels of ANGPTL4 protein in the human liver

**Figure 1:**
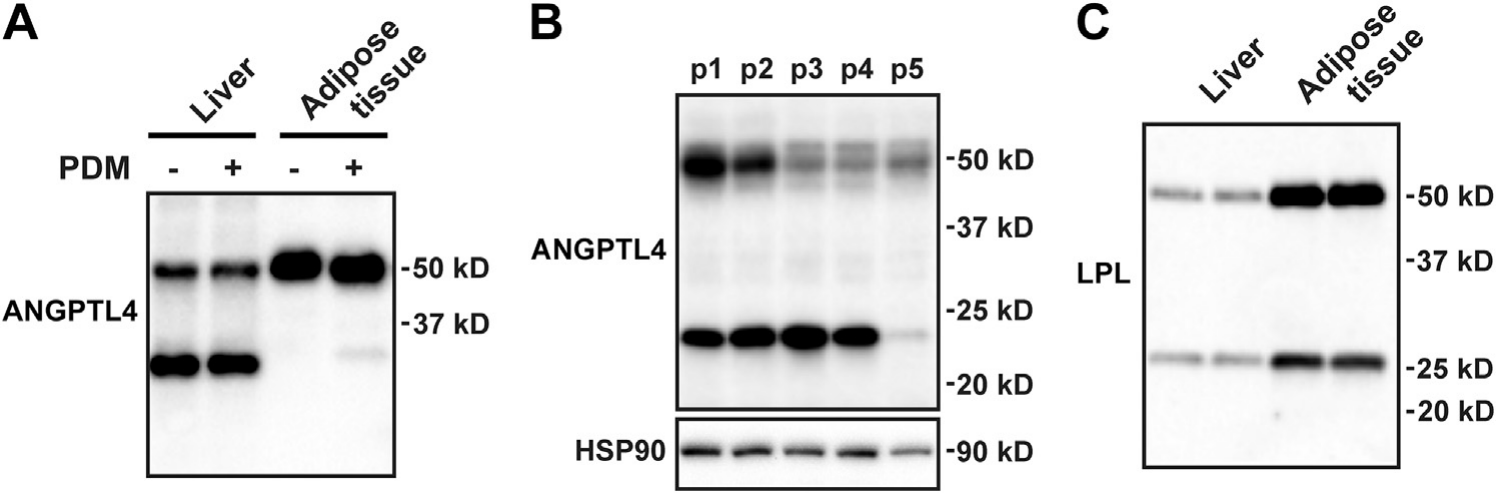
Abundance of ANGPTL4 protein in human liver. A: ANGPTL4 protein levels in cell lysates of human liver and adipose tissue treated with protein deglycosylation mix (PDM), as determined by Western blot. About 15 μg of proteins were loaded per lane. B: ANGPTL4 protein levels in the liver of five different individuals, as determined by Western blot. HSP90 was used as a loading control. C: LPL protein levels in human liver and adipose tissue as determined by Western blot. About 10 μg of proteins were loaded per lane. HSP90, heat shock protein 90.

To verify whether the liver is an appropriate target organ for ANGPTL4 silencing in humans, we determined the abundance of ANGPTL4 protein in human liver compared with adipose tissue. In contrast to mice, which exhibit higher ANGPTL4 protein and mRNA levels in adipose tissue than in the liver (8, 47), ANGPTL4 protein levels were comparable in human liver and adipose tissue (Fig. 1A). As shown previously (44), ANGPTL4 was cleaved in the human liver but not adipose tissue. As observed in mouse adipose tissue (18), the mobility of ANGPTL4 in human adipose tissue was slightly enhanced by deglycosylation. Interestingly, the absolute level of hepatic ANGPTL4 protein and the degree of ANGPTL4 cleavage were variable among different individuals (Fig. 1B). Although the *LPL* gene is very weakly expressed in the liver, we easily detected LPL protein in human liver (Fig. 1C), which likely represents endothelial-derived LPL coming from the periphery (48). A similar cleavage pattern of LPL was observed in human liver and adipose tissue (Fig. 1C). Overall, these data suggest that ANGPTL4 protein is abundantly present in human liver and thus may be a suitable target for therapeutic gene silencing.

### Identification of a mouse ANGPTL4-specific ASO

ASOs were designed against the pre-mRNA of *Angptl4* defined by the ENSEMBL transcript ID ENSMUST00000002360. About 148 *Angptl4-*specific ASOs were selected to identify the most potent and tolerated ASOs. Using cellular screens, the most promising candidate was identified, which was further characterized in this study. In vitro characterization was performed with naked non-GalNAc-modified ASOs.

**Figure 2:**
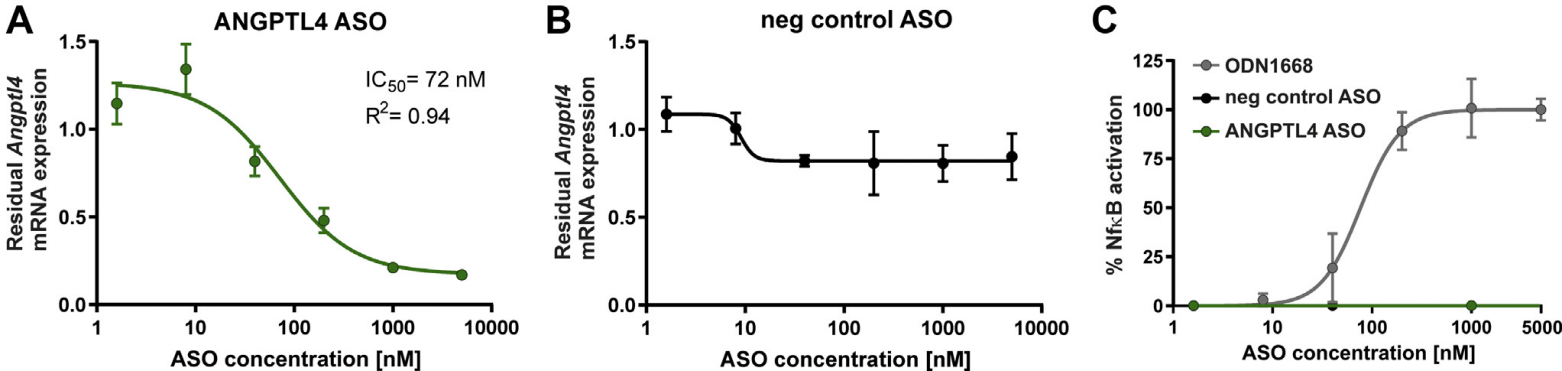
In vitro characterization of ANGPTL4-specific ASO. Measurement of the concentration response of ANGPTL4-specific ASO (A) and nontargeting control ASO (B). About 15,000 primary mouse hepatocytes/well were seeded in 96-well plates and treated with different concentrations of the respective oligonucleotide. After 3 days of treatment, cells were lysed and *Angptl4* mRNA expression was measured using the QuantiGene RNA Singleplex assay. *Angptl4* mRNA expression values were normalized to the expression of the housekeeping gene HPRT1. Residual *Angptl4* mRNA expression relative to mock-treated cells (set as 1) is shown. C: HEK293 cells expressing a mouse TLR9 NF-κB luciferase reporter plasmid were treated with the indicated concentrations of ODN1668, Neg-Ctrl, or ANGPTL4 ASO. After 20 h, the cells were treated with ONE-Glo EX reagent, and luminescence was measured at 560 nm. Values were normalized to untreated cells. ANGPTL4 ASO is indicated in green. Data are represented as the mean of triplicate wells ± SD. HEK, human embryonic kidney cell.

Concentration-dependent effects of the most potent and tolerated ANGPTL4 ASO were tested by exposing mouse primary hepatocytes to different concentrations of the ASO. Endogenous mRNA levels were evaluated after 3 days of treatment with ASO, and the 50% inhibitory concentration (IC_50_) for the inhibition of *Angptl4* expression was determined (Fig. 2A, B). The ANGPTL4 ASO was highly potent in primary hepatocytes with more than 80% knockdown of *Angptl4* mRNA at the highest concentration and an IC_50_ value of 72 nM (Fig. 2A). In contrast, the Neg-Ctrl ASO, which does not have any complementarity to any human or murine mRNA, did not reduce *Angptl4* expression levels (Fig. 2B).

Oligonucleotides can trigger immune activation leading to cytokine release. This immune activation is mediated by pattern recognition receptors, such as the TLRs. Binding of immune-stimulatory ligands, for example, bacterial DNA or immune-stimulatory oligonucleotides, with or without nonmethylated CpG dinucleotides, can result in TLR activation (41, 49). The selected ANGPTL4 ASO was therefore analyzed for its potential to activate TLR9, enabling a safety assessment for subsequent in vivo studies. However, TLR9 signaling as determined by NF-κB-activation was not induced by treatment with ANGPTL4 ASO in human embryonic kidney 293 cells stably transfected with murine TLR9 (Fig. 2C), whereas the murine TLR9 agonist ODN1668 induced a considerable concentration-dependent response.

### Short-term ANGPTL4 silencing in fed and fasted mice

**Figure 3:**
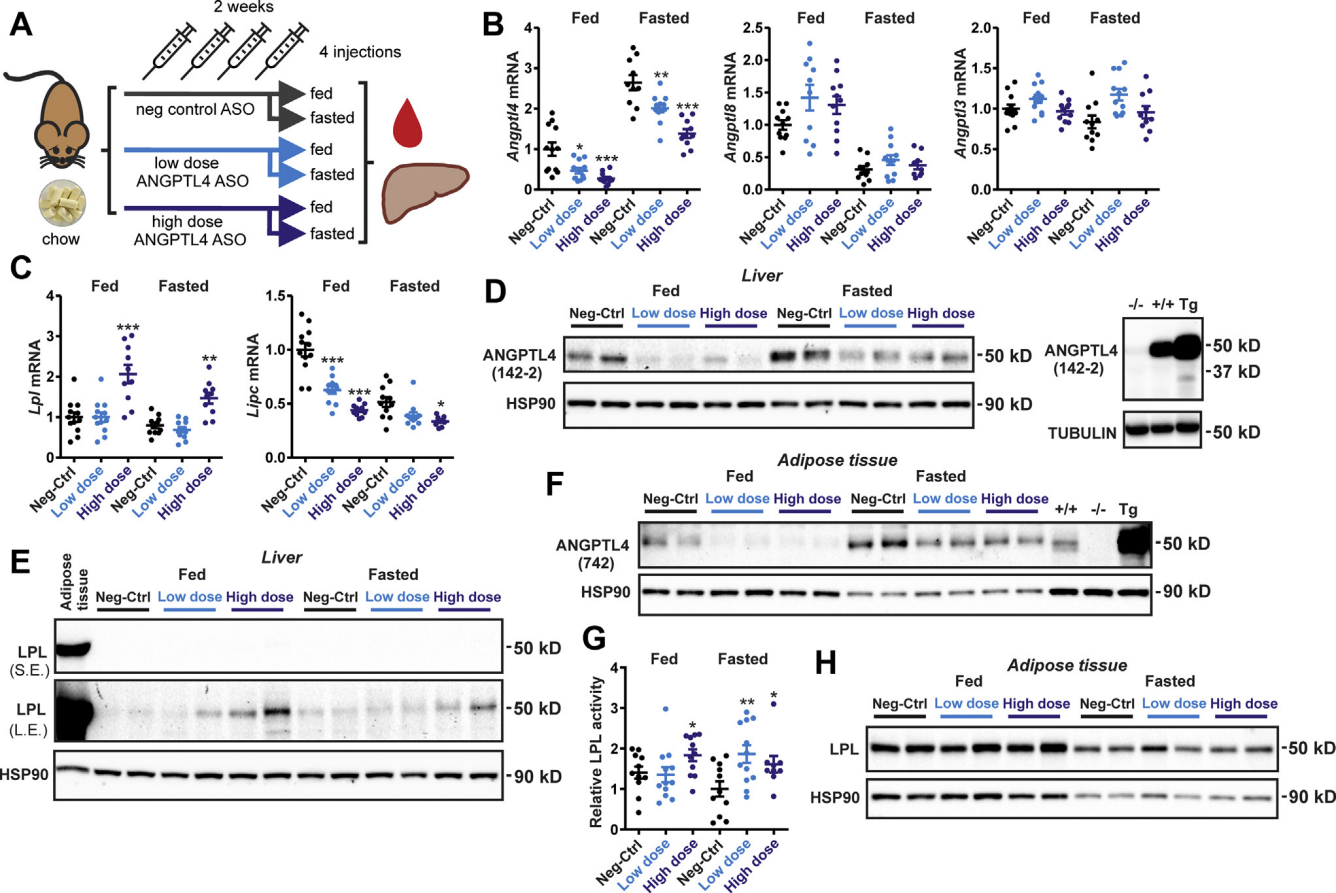
ANGPTL4 ASO reduces ANGPTL4 protein levels in mouse liver and adipose tissue. A: Schematic overview of the set-up of the study. Male C57BL/6J mice fed chow were randomly assigned to either treatment with Neg-Ctrl ASO (1.25 mg/kg, *n* = 23), low-dose ANGPTL4 ASO (0.625 mg/kg, *n* = 24), or high-dose ANGPTL4 ASO (1.25 mg/kg, *n* = 21) via subcutaneous injections twice a week for a total duration of 2 weeks. In each group, half of the mice were euthanized in the ad libitum fed state and half after a 24 h fast. B: *Angptl4*, *Angptl3*, and *Angptl8* mRNA levels in the liver as determined by quantitative PCR (qPCR). C: *Lpl* and *Lipc* mRNA levels in the liver as determined by qPCR. D: Hepatic ANGPTL4 protein levels as determined by Western blot. Right panel: Livers from wild-type, *Angptl4*^−/−^, and *Angptl4*-Tg mice were used as a reference. E: Hepatic LPL protein levels as determined by Western blot. Adipose tissue was used as a reference. L.E., long exposure; S.E., short exposure. F: Adipose tissue ANGPTL4 protein levels as determined by Western blot. Adipose tissue from wild-type, *Angptl4*^−/−^ and *Angptl4*-Tg mice was used as a reference. G: Adipose tissue LPL activity using EnzChek lipase substrate. H: Adipose tissue LPL protein levels as determined by Western blot. HSP90 was used as a loading control. In the graphs, the horizontal bar represents the mean and the error bars represent SEM. Asterisk indicates significantly different from Neg-Ctrl ASO according to Tukey’s post hoc test. ∗*P* < 0.05, ∗∗*P* < 0.01, and ∗∗∗*P* < 0.001. HSP90, heat shock protein 90.

To test the efficacy of ANGPTL4 silencing in lowering plasma TG levels in vivo, we performed injections of the ANGPTL4 ASO in mice. Previous studies have shown that whole-body ANGPTL4 overexpression or deletion mainly affects plasma lipid levels in the fasted state and not or only minimally in the fed state (32, 50, 51, 52). Accordingly, we hypothesized that the effect of ANGPTL4 silencing on plasma lipids would also mainly be evident in the fasted state. To verify this notion, we injected wild-type mice twice a week for 2 weeks with 1.25 mg/kg ANGPTL4 ASO (high dose), 0.625 mg/kg ANGPTL4 ASO (low dose), or 1.25 mg/kg Neg-Ctrl ASO (control) and euthanized the mice either in the ad libitum fed state or after a 24 h fast (Fig. 3A). Fasting resulted in a marked increase in hepatic *Angptl4* mRNA (Fig. 3B). Compared with the mice treated with Neg-Ctrl ASO, the mice that received low-dose or high-dose ANGPTL4 ASO had 53% or 72% lower hepatic *Angptl4* mRNA levels in the fed state, respectively, and 24% and 48% lower *Angptl4* mRNA in the fasted state (Fig. 3B). No differences in *Angptl8* or *Angptl3* mRNA levels were observed among the different ASO groups in either the fed or fasted state (Fig. 3B). Interestingly, *Lpl* mRNA levels were increased in the fed and fasted mice that received the high-dose ANGPTL4 ASO compared with Neg-Ctrl ASO, whereas *Lipc* mRNA—encoding hepatic lipase—was dose-dependently decreased in the ANGPTL4 ASO mice (Fig. 3C). Mirroring the decrease in *Angptl4* mRNA, hepatic protein levels of ANGPTL4 were markedly reduced by the treatment with ANGPTL4 ASO (Fig. 3D), whereas hepatic LPL protein levels, although hardly detectable and much lower than in adipose tissue, were higher in the mice treated with ANGPTL4 ASO (Fig. 3E). Unexpectedly, ANGPTL4 ASO treatment also led to a pronounced decrease in ANGPTL4 protein levels in adipose tissue (Fig. 3F), which was accompanied by increased adipose LPL activity (Fig. 3G). By contrast, LPL protein levels in adipose tissue, while being reduced by fasting, were not affected by ANGPTL4 ASO (Fig. 3H). Taken together, treatment with ANGPTL4 ASO effectively decreased ANGPTL4 protein levels in the liver and adipose tissue and significantly increased adipose LPL activity.

**Figure 4:**
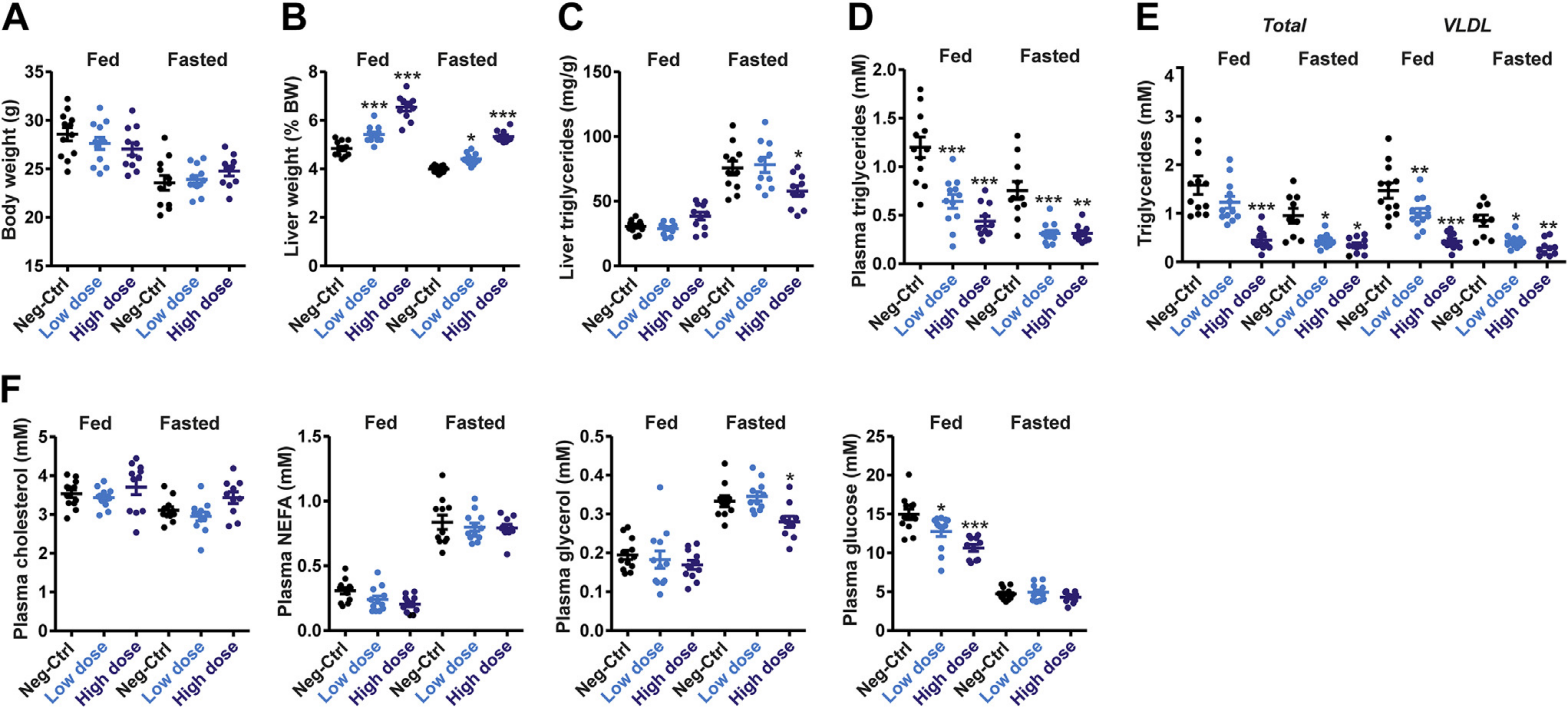
ANGPTL4 ASO reduces plasma TGs independent of feeding status. A: Bodyweight. B: Liver weight. C: Liver TGs. D: Plasma TGs. E: HPLC-based plasma lipoprotein profiling. F: Plasma levels of cholesterol, nonesterified fatty acids, glycerol, and glucose. In the graphs, the horizontal bar represents the mean and the error bars represent SEM. Asterisk indicates significantly different from Neg-Ctrl ASO according to Tukey’s post hoc test. ∗*P* < 0.05, ∗∗*P* < 0.01, and ∗∗∗*P* < 0.001.

Additional analyses showed that body weight was lower in the fasted mice than in the fed mice but was not different among the treatment groups (Fig. 4A). By contrast, liver weight was 11% and 34% higher in the low-dose and high-dose ANGPTL4 ASO groups compared with Neg-Ctrl ASO, respectively, which was very similar in the fed and fasted mice (Fig. 4B). As expected, TG levels in the liver were increased by fasting (Fig. 4C). Interestingly, in fasted mice, liver TG levels were significantly reduced in the mice treated with high-dose ANGPTL4 ASO (Fig. 4C). Consistent with the plasma TG-raising effect of ANGPTL4, plasma TG levels were significantly decreased in the ANGPTL4 ASO mice. Specifically, compared with the Neg-Ctrl ASO group, mice that received low-dose or high-dose ANGPTL4 ASO showed 46% or 63% lower plasma TG levels in the fed state, respectively, and 59% and 58% lower plasma TG levels in the fasted state (Fig. 4D). Plasma lipoprotein profiling by HPLC confirmed these findings and showed that the lower plasma TG levels are accounted for by lower levels of VLDL-TG (Fig. 4E). By contrast, plasma cholesterol levels were similar in the three ASO groups (Fig. 4F). Plasma nonesterified fatty acids were markedly elevated in the fasted mice compared with the fed mice, but no significant differences were detected among the three ASO groups, while plasma glycerol levels were lower in the high-dose ANGPTL4 ASO mice (Fig. 4F). Remarkably, plasma glucose levels in the fed state were significantly lower in the mice treated with ANGPTL4 ASO compared with the Neg-Ctrl ASO mice (Fig. 4F). Overall, these data indicate that ANGPTL4 silencing by ASOs effectively reduced plasma TG levels. However, in contrast to our hypothesis, the magnitude of the decrease in plasma TG by ANGPTL4 ASO was similar in the fed and fasted state.

### Long-term ANGPTL4 silencing in mice fed a HFD

**Figure 5:**
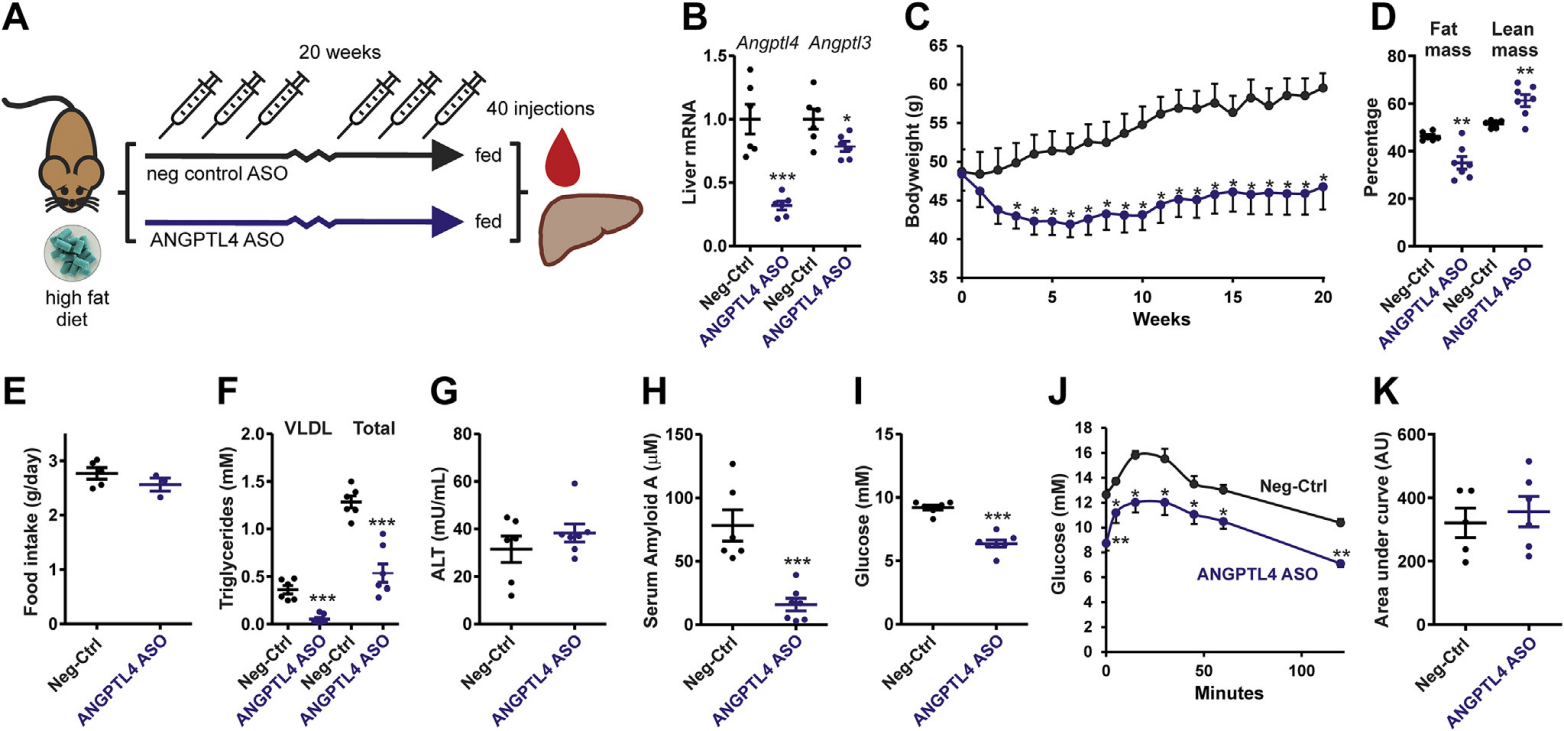
Pilot study on the effect of ANGPTL4 ASO in diet-induced obese mice. A: Schematic overview of the set-up of the study. Male C57BL/6J mice were randomly assigned to either treatment with Neg-Ctrl ASO (1.25 mg/kg, *n* = 6) or ANGPTL4 ASO (1.25 mg/kg, *n* = 7) via subcutaneous injections twice a week for a total duration of 20 weeks. During the 20 weeks, mice were given a HFD (60 en% fat). B: *Angptl4* and *Angptl3* mRNA levels in the liver as determined by quantitative PCR (qPCR). C: Bodyweight change from the start of the ASO and HFD treatment. D: Relative fat and lean mass as determined by echo MRI. E: Average food intake determined per cage but expressed per mouse during the 20 weeks. F: Plasma TG concentration. All plasma measurements were after a 6 h fast. G: Plasma alanine aminotransferase activity. H: Serum amyloid A level in plasma. I: Plasma glucose concentration. J: Intraperitoneal glucose tolerance test after a 6 h fast. K: Area under the curve for the glucose tolerance test corrected for baseline glucose levels. The horizontal bar represents the mean, and the error bars represent SEM. Asterisk indicates significantly different from Neg-Ctrl ASO according to Student’s *t*-test. ∗*P* < 0.05, ∗∗*P* < 0.01, and ∗∗∗*P* < 0.001.

To investigate the long-term safety of subcutaneous administration of ANGPTL4 ASO, we performed two independent studies at two different locations in mice fed a HFD. In the first pilot study, mice were given 1.25 mg/kg ANGPTL4 ASO or Neg-Ctrl ASO twice a week for 20 weeks while being fed a HFD containing 60 energy% fat (Fig. 5A). ANGPTL4 ASO treatment led to a 68% decrease in hepatic *Angptl4* mRNA levels and a 21% decrease in *Angptl3* mRNA (Fig. 5B). Whereas the Neg-Ctrl ASO mice steadily gained weight on the HFD, the ANGPTL4 ASO mice lost weight in the first 5 weeks, followed by a slow recovery thereafter (Fig. 5C). The lower body weight in the ANGPTL4 ASO mice was accompanied by a lower relative fat mass and a higher relative lean mass (Fig. 5D). No significant differences in food intake were found between the ANGPTL4 and Neg-Ctrl ASO mice (Fig. 5E). In agreement with the plasma TG-raising effect of ANGPTL4, plasma VLDL-TG and total TG levels were markedly lower in the ANGPTL4 ASO mice compared with Neg-Ctrl ASO mice (Fig. 5F). Plasma alanine aminotransferase levels were not significantly different between the ANGPTL4 and Neg-Ctrl ASO mice (Fig. 5G), whereas serum amyloid A levels in plasma were significantly lower in the ANGPTL4 ASO mice compared with Neg-Ctrl ASO mice (Fig. 5H). Consistent with the lower body weight and fat mass, ANGPTL4 ASO mice exhibited lower fasting plasma glucose levels (Fig. 5I) and had lower glucose levels during the intraperitoneal glucose tolerance test than the Neg-Ctrl ASO mice (Fig. 5J). However, no difference in area under the curve (corrected for baseline glucose) was observed between the ANGPTL4 and Neg-Ctrl ASO mice (Fig. 5K). Importantly, no signs of ascites or lymphadenopathy were observed in any of the mice. Collectively, these data suggest that chronic ANGPTL4 inactivation via ASOs is safe, despite the minor increase in plasma alanine aminotransferase, and confers a metabolic benefit by lowering plasma TG and glucose levels.

**Figure 6:**
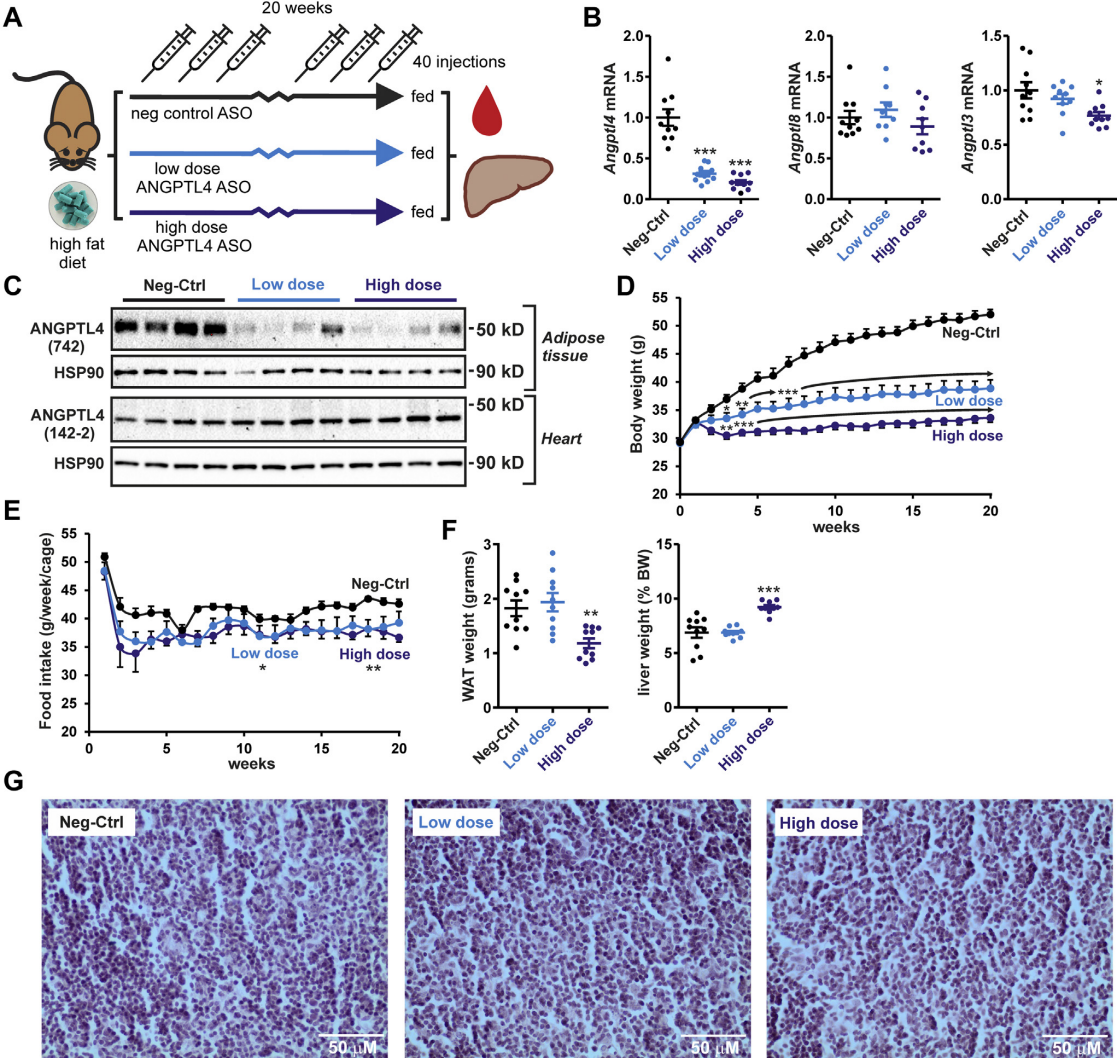
ANGPTL4 ASO reduces food intake and body weight gain in diet-induced obese mice. A: Schematic overview of the set-up of the study. Male C57BL/6J mice fed a HFD (45 en% fat) were randomly assigned to either treatment with Neg-Ctrl ASO (1.25 mg/kg, *n* = 10), low-dose ANGPTL4 ASO (0.625 mg/kg, *n* = 10), or high-dose ANGPTL4 ASO (1.25 mg/kg, *n* = 10) via subcutaneous injections twice a week for a total duration of 20 weeks. B: *Angptl4*, *Angptl3*, and *Angptl8* mRNA levels in the liver as determined by quantitative PCR (qPCR). C: ANGPTL4 protein levels in the adipose tissue and heart as determined by Western blot. HSP90 was used as a loading control. D: Bodyweight change from the start of the ASO and HFD treatment. E: Average food intake determined per cage (two mice/cage) during the 20 weeks. Mean food intake per cage across 20 weeks was significantly lower in the low- and high-dose ANGPTL4 ASO groups compared with the Neg-Ctrl ASO group (*P* < 0.05 and *P* < 0.01, respectively, indicated by an asterisk). F: Weight of the epididymal fat pad (absolute weight) and the liver (percent of body weight). G: Representative hematoxylin and eosin staining of the mesenteric lymph nodes. In the graphs, the horizontal bar represents the mean and the error bars represent SEM. Asterisk indicates significantly different from Neg-Ctrl ASO according to Tukey’s post hoc test. ∗*P* < 0.05, ∗∗*P* < 0.01, and ∗∗∗*P* < 0.001. HSP90, heat shock protein 90.

In a second more comprehensive study, mice were given either 1.25 mg/kg ANGPTL4 ASO (high dose), 0.625 mg/kg ANGPTL4 ASO (low dose), or 1.25 mg/kg Neg-Ctrl ASO twice a week for 20 weeks while being fed a HFD containing 45 energy% fat (Fig. 6A). Compared with the mice treated with Neg-Ctrl ASO, mice treated with low-dose or high-dose ANGPTL4 ASO showed 69% or 80% lower hepatic *Angptl4* mRNA levels, respectively, indicating that ANGPTL4 ASO effectively downregulated hepatic *Angptl4* expression (Fig. 6B). *Angptl8* mRNA levels were not different among the groups, whereas *Angptl3* mRNA was significantly lower in the mice that received the high-dose ANGPTL4 ASO (Fig. 6B). Western blot confirmed the downregulation of ANGPTL4 protein by ANGPTL4 ASO in the adipose tissue but not in the heart (Fig. 6C). As previously observed (47), ANGPTL4 protein moved at a slightly lower molecular weight in the heart than in adipose tissue.

Remarkably, mice treated with the ANGPTL4 ASO gained much less weight, which was most pronounced for the high-dose group (Fig. 6D). Food intake was significantly lower in the ANGPTL4 ASO mice compared with the Neg-Ctrl ASO mice (Fig. 6E). No significant differences in food intake were observed between the low-dose and high-dose ANGPTL4 ASO groups. The weight of the epididymal fat depot was significantly lower in the high-dose ANGPTL4 ASO group than in the other two groups, whereas the relative liver weight was significantly higher in the high-dose group than in the other two groups (Fig. 6F). In line with the results from the pilot study, no ascites was observed in any of the mice. The mesenteric lymph nodes were also not visually enlarged in any of the mice, did not contain any Touton giant cells, and were histologically normal (Fig. 6G).

**Figure 7:**
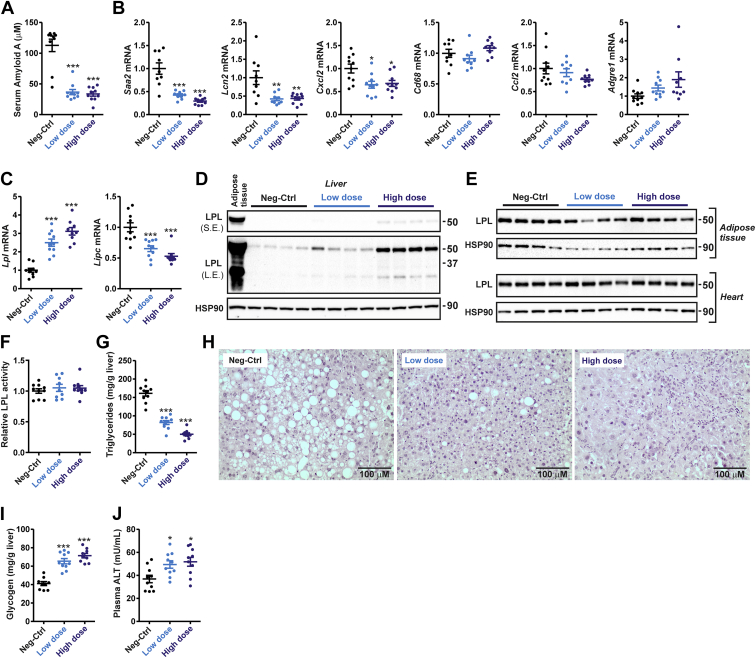
ANGPTL4 ASO reduces steatosis and increases liver LPL content in diet-induced obese mice. Male C57BL/6J mice fed a HFD (45 en% fat) were randomly assigned to either treatment with Neg-Ctrl ASO (1.25 mg/kg, *n* = 10), low-dose ANGPTL4 ASO (0.625 mg/kg, *n* = 10), or high-dose ANGPTL4 ASO (1.25 mg/kg, *n* = 10) via subcutaneous injections twice a week for a total duration of 20 weeks. A: Serum amyloid A level in plasma. B: Hepatic mRNA levels of inflammatory markers as determined by quantitative PCR (qPCR). C: Hepatic mRNA levels of *Lpl* and *Lipc*. D: Hepatic LPL protein levels as determined by Western blot. Adipose tissue was used as a reference. HSP90 was used as a loading control. E: LPL protein levels in the adipose tissue and heart as determined by Western blot. HSP90 was used as a loading control. F: Adipose tissue LPL activity using EnzChek lipase substrate. G: Liver TG levels. H: Representative hematoxylin and eosin staining of the liver. I: Liver glycogen levels. J: Plasma alanine aminotransferase activity. In the graphs, the horizontal bar represents the mean and the error bars represent SEM. Asterisk indicates significantly different from Neg-Ctrl ASO according to Tukey’s post hoc test. ∗*P* < 0.05, ∗∗*P* < 0.01, and ∗∗∗*P* < 0.001. HSP90, heat shock protein 90.

As observed in the pilot HFD study, serum amyloid A levels in plasma were markedly lower in the ANGPTL4 ASO mice compared with the Neg-Ctrl ASO mice (Fig. 7A). Similarly, liver mRNA levels of *Saa2*, *Lcn2*, and *Cxcl2* were significantly lower in the ANGPTL4 ASO mice (Fig. 7B). Expression of *Ccl2* and macrophage marker *Cd68* was not different among the groups, whereas expression of macrophage marker *Adgre1* tended to increase in the ANGPTL4 ASO groups (Fig. 7B). As observed in the fed and fasted mice, the hepatic expression of *Lipc* was markedly decreased by ANGPTL4 ASO, whereas *Lpl* expression was significantly increased (Fig. 7C). Consistent with the *Lpl* mRNA data, hepatic LPL protein levels, despite being much lower than in adipose tissue, were increased in the ANGPTL4 ASO groups (Fig. 7D). LPL protein levels in adipose tissue and the heart were not affected by ANGPTL4 ASO (Fig. 7E). Also, adipose LPL activity was not significantly different among the groups (Fig. 7F). Biochemical analysis showed a marked decrease in liver TG levels in the ANGPTL4 ASO mice compared with the Neg-Ctrl ASO mice (Fig. 7G). Histological examination of livers stained by hematoxylin and eosin confirmed the markedly reduced liver lipid content in the mice treated with ANGPTL4 ASO and revealed the presence of mild focal inflammatory infiltrates (Fig. 7H). Interestingly, liver glycogen levels were markedly increased in the ANGPTL4 ASO groups (Fig. 7I). Additionally, plasma alanine aminotransferase levels were modestly but statistically significantly higher in the ANGPTL4 ASO mice than in the Neg-Ctrl ASO mice (Fig. 7J), suggesting a modest increase in liver damage. Collectively, these data demonstrate that chronic treatment of mice with ANGPTL4 ASO in combination with a HFD leads to minor damage and inflammation in mouse liver but does not lead to the macroscopic, microscopic, and biochemical perturbations observed in whole-body ANGPTL4-null mice (27).

**Figure 8:**
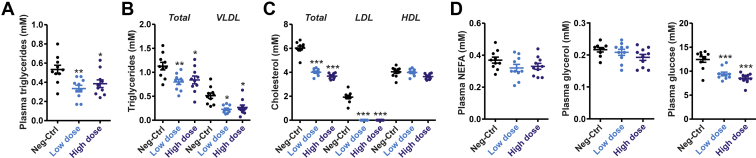
ANGPTL4 ASO reduces plasma lipids in diet-induced obese mice. Male C57BL/6J mice fed a HFD (45 en% fat) were randomly assigned to either treatment with Neg-Ctrl ASO (1.25 mg/kg, *n* = 10), low-dose ANGPTL4 ASO (0.625 mg/kg, *n* = 10), or high-dose ANGPTL4 ASO (1.25 mg/kg, *n* = 10) via subcutaneous injections twice a week for a total duration of 20 weeks. A: Plasma TGs. B: Plasma total TG and VLDL-TG levels as determined by HPLC-based lipoprotein profiling. C: Plasma total cholesterol, LDL-cholesterol, and HDL-cholesterol as determined by HPLC-based lipoprotein profiling. D: Plasma levels of nonesterified fatty acids, glycerol, and glucose. The horizontal bar represents the mean, and the error bars represent SEM. Asterisk indicates significantly different from Neg-Ctrl ASO according to Tukey’s post hoc test. ∗*P* < 0.05, ∗∗*P* < 0.01, and ∗∗∗*P* < 0.001.

We next measured the plasma levels of various metabolites. Compared with the control group, plasma TG levels were 37% and 28% lower in the low-dose and high-dose ANGPTL4 ASO groups, respectively (Fig. 8A). Lipoprotein profiling by HPLC supported these data by showing significantly lower total TG and VLDL-TG levels in the ANGPTL4 ASO mice compared with the Neg-Ctrl ASO mice (Fig. 8B). Total cholesterol levels were also significantly lower in the ANGPTL4 ASO mice compared with Neg-Ctrl ASO mice, which was accounted for by significantly lower plasma LDL-C levels (Fig. 8C). No differences were observed among the groups for plasma nonesterified fatty acids and glycerol, whereas plasma glucose levels were markedly lower in the ANGPTL4 ASO mice than in the Neg-Ctrl ASO mice (Fig. 8D). Taken together, these data show that treatment of mice fed a HFD with ANGPTL4 ASO leads to favorable metabolic changes, including lower plasma TG, lower plasma cholesterol, and lower plasma glucose levels, as well as lower hepatic TG content.

## Discussion

ANGPTL4 is abundant in the human liver, where it could be targeted pharmacologically to lower plasma lipids. Here, we aimed to study the efficacy and long-term safety of liver-specific silencing of ANGPTL4 in mice using GalNAc-conjugated ASOs. We found that two injections per week of two different doses of ANGPTL4 ASO effectively reduced hepatic ANGPTL4 levels, plasma TG, and plasma glucose levels in mice fed a HFD for 20 weeks. Importantly, chronic treatment with ANGPTL4 ASO did not induce any of the macroscopic and microscopic abnormalities observed in whole-body ANGPTL4-null mice fed a HFD, including mesenteric lymphadenopathy, a massive acute phase response, and chylous ascites. Treatment of mice fed a HFD with ANGPTL4 ASO was associated with a marked reduction in weight gain, which was likely explained by a significant reduction in food intake. In mice fed chow, short-term treatment with two different doses of ANGPTL4 ASO also effectively reduced plasma TG levels in the fed and 24 h fasted state and reduced plasma glucose levels in the fed state. Overall, these data indicate that ASOs targeting ANGPTL4 effectively reduce plasma TG and glucose levels in mice without raising major safety concerns.

Based on previous studies conducted in mice and monkeys (22, 29), whole-body inactivation of ANGPTL4 using ANGPTL4-inactivating antibodies may be considered a risky strategy for lowering plasma TG levels and reducing cardiovascular risk in humans. Our data indicate that in contrast to whole-body ANGPTL4 inactivation, ANGPTL4 silencing in mice using GalNAc-conjugated ASOs does not lead to mesenteric lymphadenopathy, an acute phase response, and chylous ascites, yet still very effectively reduces plasma TG levels. Based on the data presented here, further studies in monkeys and humans on the safety and effectiveness of ANGPTL4 ASO are warranted.

Several arguments led us to hypothesize that the effect of ANGPTL4 silencing would mainly be evident in the fasted state. First, the expression of ANGPTL4 in the liver is markedly induced by fasting (15). Second, the effect of hepatocyte-specific ANGPTL4 deficiency on plasma TG levels is most pronounced during prolonged fasting (33, 34). Third, data from transgenic and whole-body ANGPTL4-null mice indicate that ANGPTL4 primarily plays a role in plasma lipid metabolism during fasting (32, 50, 51, 52). Treatment with ANGPTL4 ASO, however, was similarly effective in reducing plasma TG levels in fed mice and 24 h fasted mice. The reason for this unexpected finding remains unclear. There is no evidence that ANGPTL4 silencing in fed mice may also have impacted hepatic *Angptl3* or *Angptl8* mRNA, thereby causing additional TG lowering. Our data raise the possibility that the effect of ANGPTL4 ASO on plasma lipids in humans may be relatively unaffected by the nutritional status.

In mice fed a HFD, ANGPTL4 ASO treatment did not influence the increase in blood glucose following intraperitoneal glucose injection. However, treatment with ANGPTL4 ASO significantly lowered nonfasting plasma glucose levels in mice fed a HFD and in mice fed chow. In mice fed a HFD, we cannot exclude that the reduction in plasma glucose levels by ANGPTL4 ASO is secondary to reduced adiposity. In chow-fed mice, however, treatment with ANGPTL4 ASO was not accompanied by any change in body weight, suggesting a direct role of ANGPTL4 in regulating glucose homeostasis. Previous studies in adipocyte-specific ANGPTL4-deficient mice, whole-body ANGPTL4-null mice, and whole-body ANGPTL4-transgenic mice, all point to a stimulatory effect of ANGPTL4 on plasma glucose levels (30, 53, 54). Consistent with this notion, the carrier status of the E40K loss-of-function variant is associated with a reduction in the odds of type 2 diabetes varying from 9% to 38% (23, 24, 55, 56). Collectively, the data suggest that ANGPTL4 ASO may not only be useful for treating hypertriglyceridemia but could also be valuable in the therapeutic management of type 2 diabetes. Further mechanistic studies are necessary to better understand the link between ANGPTL4 and glucose homeostasis.

Treatment with ANGPTL4 ASO markedly reduced plasma TG levels, which can be explained by several different mechanisms. First, ANGPTL4 ASO may reduce plasma TG levels by decreasing ANGPTL4 production in the liver, which might lead to enhanced adipose LPL activity via an endocrine action of ANGPTL4. Second, ANGPTL4 ASO may lower plasma TG levels by downregulating ANGPTL4 protein in the adipose tissue, resulting in enhanced adipose LPL activity. It has been shown that ANGPTL4 deficiency in adipose tissue is associated with increased adipose LPL activity, lower plasma TG levels, and increased uptake of plasma TG into adipose tissue (30, 31). Third, ANGPTL4 ASO may reduce plasma TG levels by increasing LPL mRNA and protein levels in the liver. Singh *et al.* (33) found that ANGPTL4 ASO also enhanced hepatic LPL activity. There is evidence that liver LPL contributes to the regulation of plasma TG levels. Specifically, using two different gene targeting approaches, Liu *et al.* observed that hepatic LPL deficiency modestly increased plasma TG levels and decreased postheparin LPL activity in mice (57). In our study, although ANGPTL4 ASO increased hepatic LPL protein, the very low absolute levels of LPL protein in mouse liver compared with adipose tissue render a role of hepatic LPL in the reduction in plasma TG levels by ANGPTL4 ASO very unlikely. In addition, treatment with low-dose ANGPTL4 ASO reduced plasma TG levels in fed and fasted mice despite no change in hepatic LPL protein and mRNA levels. A fourth and final explanation for the TG-lowering effect of ANGPTL4 ASO is via the enhanced activity of hepatic lipase (33). Consistent with this notion, hepatic lipase activity was higher in liver and primary hepatocytes of hepatocyte-specific ANGPTL4-deficient mice compared with control mice (33). We tried to measure hepatic lipase protein levels by Western blot but unfortunately were unable to get trustworthy results.

As indicated above, treatment with ANGPTL4 ASO not only silenced hepatic ANGPTL4 but also substantially lowered ANGPTL4 protein levels in adipose tissue, despite the ASO being GalNAc conjugated, which is aimed at directing it to the liver. Hence, treatment with ANGPTL4 ASO cannot be considered a model for liver-specific ANGPTL4 silencing, at least in mice (33). Interestingly, the effect of ANGPTL4 ASO on adipose tissue ANGPTL4 protein levels was more pronounced in the fed state than in the fasted state. It can be speculated that this is due to the higher ratio of ANGPTL4 ASO relative to *Angptl4* mRNA in the fed state. In contrast to our observations in whole-body ANGPTL4-null mice (17, 28), LPL protein levels were not affected in the adipose tissue of mice treated with ANGPTL4 ASO, possibly because the magnitude of the decrease in ANGPTL4 protein was insufficient.

It can be noted that in comparison to heterozygous and homozygous ANGPTL4-null mice, the magnitude of plasma TG reduction by ANGPTL4 ASO appears to be disproportional to the relative decrease in ANGPTL4 protein in liver and adipose tissue. However, an important difference between ANGPTL4-null mice and mice treated with ANGPTL4 ASO is that the former lack ANGPTL4 since conception. It is conceivable that the early deficiency of ANGPTL4 triggers specific compensatory mechanisms that are not activated when ANGPTL4 is silenced during adulthood.

Our data largely confirm previous studies by Singh *et al.* (33), who found that treatment of chow-fed mice with ANGPTL4 ASO resulted in decreased plasma TG, total cholesterol, HDL-C, and glucose levels. Also, consistent with our data, Singh did not detect any gut inflammation, mesenteric lymphadenopathy, and chylous ascites in mice treated with ANGPTL4 ASO when combined with chronic HFD feeding. ANGPTL4 ASO-treated mice were protected from HFD-induced obesity and had improved glucose tolerance and insulin sensitivity and lower circulating levels of serum amyloid A. However, in contrast to Singh, we found significantly lower food intake in mice treated with ANGPTL4 ASO compared with Neg-Ctrl ASO, both at higher and lower doses (33). Theoretically, the ∼10% reduction in food intake in the ANGPTL4 ASO-treated mice could account for the reduced body weight gain in these animals.

Currently, we do not have a mechanistic explanation for the lower food intake in the ANGPTL4 ASO-treated mice. In previous studies, we did not see any change in food intake in whole-body ANGPTL4-null mice fed either a low-fat diet or HFD, at least before the development of inflammatory complications (27, 53, 58). Paradoxically, others observed increased food intake in ANGPTL4-null mice following a fast, as well as diminished anorectic responses to leptin, insulin, and glucose, suggesting that ANGPTL4 suppresses food intake (59). An important difference between the ANGPTL4-null mice and mice treated with ANGPTL4 ASO is that the former lack ANGPTL4 since conception. It is conceivable that hepatic ANGPTL4 promotes food intake, but this role may not be evident when studying ANGPTL4-null mice either because ANGPTL4 is lacking from conception or because the effect of hepatic ANGPTL4 is counteracted by ANGPTL4 produced in other tissues.

Our study also has limitations, one of which is that we used male mice only. Accordingly, we cannot draw any conclusion about the ability of ANGPTL4 ASO to lower plasma TG levels in female mice. In the first study using whole-body ANGPTL4-null mice, plasma TG levels were more strongly reduced in male mice than in female mice (51). It might thus be expected that ANGPTL4 ASO may be less effective in reducing plasma TG levels in female mice than in male mice. However, in a more recent study, plasma TG levels were reduced to a similar extent by adipocyte-specific ANGPTL4 deficiency in male and female mice (31).

Intriguingly, the weights of the livers were elevated in the ANGPTL4 ASO-treated mice. Currently, we do not have a good explanation for this finding. Whole-body ANGPTL4-null mice have normal liver weights, suggesting that the higher liver weights in ANGPTL4 ASO-treated mice are not related to the reduction in ANGPTL4 levels. In contrast to our data, Singh observed lower liver weights in the mice treated with ANGPTL4 ASO (33). The reason for this discrepancy is unclear.

In conclusion, we find that silencing of ANGPTL4 using ASOs effectively decreases plasma TG and glucose levels in male mice fed chow or a HFD without causing any of the inflammatory complications observed in whole-body ANGPTL4-null mice or mice treated with anti-ANGPTL4 monoclonal antibodies. These results pave the way for the potential future application of ANGPTL4 ASO in plasma lipid management in humans.

## Data availability

All data are contained within the article.

## Conflict of interest

A. S., S. M., and F. J. are employees of Secarna Pharmaceuticals. E. K., M. M. J., and S. K. N. are employees of Lipigon Pharmaceuticals. S. K. is a paid consultant for Lipigon. All other authors declare that they have no conflicts of interest with the contents of this article.
